# Visualizing Colonoscopy Capacity for Public Health Use

**DOI:** 10.5888/pcd15.170421

**Published:** 2018-06-07

**Authors:** Milan H. Vu, Jesse L. Tran

**Affiliations:** 1 Centers for Disease Control and Prevention, Atlanta, Georgia; 2 North Dakota Department of Health, Bismarck, North Dakota

**Figure:**
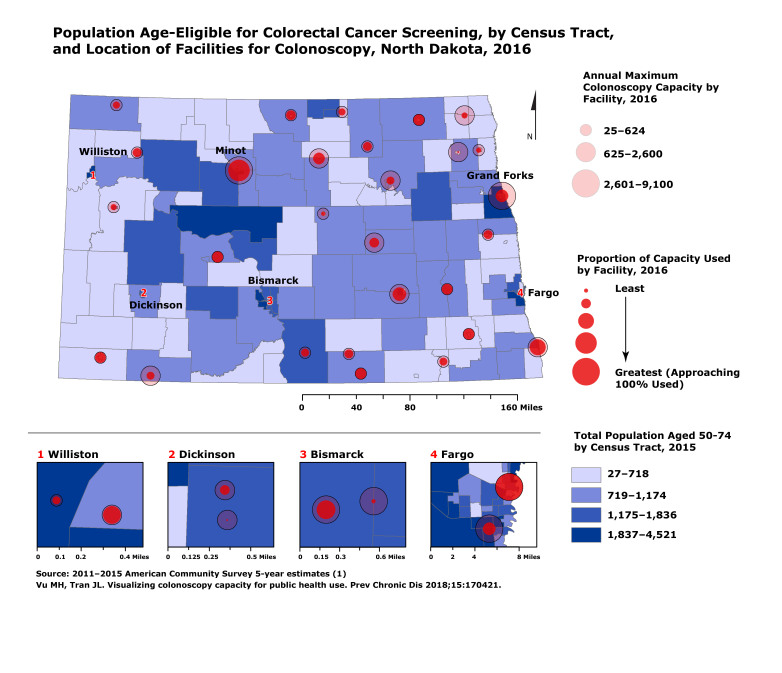
The map depicts locations of facilities performing colonoscopy in North Dakota, in addition to each facility’s maximum annual colonoscopy capacity and proportion of capacity used. Overall, 60.7% of the statewide capacity is used. The distribution of North Dakota’s age-eligible population for colorectal cancer screening is shown by census tract. This type of data collection and visualization is appropriate for informing and generating discussion among stakeholders around health status, needs, and gaps.

## Background

In North Dakota, colorectal cancer (CRC) is the third most commonly diagnosed type of cancer and ranks second in late-stage diagnosis among all cancers (2). The public health community has prioritized improving colorectal cancer screening rates in North Dakota, which are currently at 64.7% (3). As a result, it is important to use and effectively communicate data in the process of developing strategies and making decisions.

The US Preventive Services Task Force recommends CRC screening start at age 50 and continue to age 75 for those of average risk (4). Despite the availability of several high-quality testing options, colonoscopy accounts for about 80% of CRC screening in North Dakota (3,5). Mapping the location of facilities performing colonoscopy, each facility’s capacity, and the age-eligible population for CRC screening aids stakeholders in understanding population distribution, service availability, and opportunities for interventions to increase CRC screening rates; for example, expanded use of alternative screening modalities such as fecal immunochemical testing (FIT).

## Methods

The age-eligible population for CRC screening, defined as the total population aged 50 to 74 years, was obtained by census tract from the US Census Bureau’s American Community Survey (ACS) (1). A telephone survey of ambulatory surgical centers and hospitals was conducted in March of 2016 to attain measures for used and maximum colonoscopy capacity. Facilities were identified by using the North Dakota Department of Health’s facilities directory for ambulatory surgical centers and hospitals. Of the 11 ambulatory surgical centers listed, 6 were excluded from the survey because they exclusively focused on services unrelated to colonoscopy (eg, cosmetic, ophthalmologic, or orthopedic surgery). Of the 55 hospitals listed, 7 were excluded from the survey by the same criterion (ie, services unrelated to colonoscopy).

At each facility, staff (eg, directors of nursing services, directors of surgical services, business office) were asked a series of questions, via telephone survey, regarding the facility’s colonoscopy services. Survey questions were adapted from the 2012 Survey of Endoscopic Capacity (SECAP), and centered around how many colonoscopies were performed and how many additional colonoscopies could be performed within a given time (5). Because facilities provided various time frames, ranging from weekly to annual estimates, when reporting the number of colonoscopies performed, all measures were scaled to an annual timeframe to allow for comparison. From this, maximum capacity was defined as the number of colonoscopies performed plus the number of additional colonoscopies that could be performed per year at a given facility. Proportion of capacity used was calculated by dividing the number of colonoscopies performed by the maximum capacity.

ArcGIS version 10.3.1 (Esri) for desktop was used to assemble ACS population data and colonoscopy capacity data, apply a GIS address geocoder to plot addresses of North Dakota facilities, and generate the map. Numbered insets are included where facilities are close to each other and would otherwise overlap when depicted on the state map.

## Main Findings

The map shows relative capacity of each facility to perform colonoscopy. Annual maximum capacity of colonoscopies per year ranged from 25 to 9,100 colonoscopies. Additionally, the proportion of the maximum capacity used at each facility ranged from 13.8% to 100%. Six facilities reported being at 100% capacity.

In addition to capacity, there are differences in the distribution of facilities across the state. If we split the state in half, including Bismarck and everything west, there are roughly half as many facilities that perform colonoscopy in that area as compared with the eastern half of the state. This coincides with the much more rural and lower population density in the western part of the state.

In terms of population distribution, most of the age-eligible population is near the major cities. Although this population display is limited to the average risk, age-eligible population for CRC screening, a more accurate proxy for population need would integrate factors that affect the actual need and demand for these services, such as prior screening and personal risk factors. Rather, this visualization is intended to be a starting point of reference for stakeholder discussion.

While it is not surprising that facilities offering colonoscopy services are in more highly populated counties, this map highlights differences in distribution between various parts of the state — east and west, urban and rural. Travel to a facility that offers colonoscopy, or one that has adequate availability, introduces additional barriers including time off from work, lost wages, food and lodging, and transportation. With the availability of alternative tests to colonoscopy for CRC screening, this might point to opportunities for increased use and uptake of stool-based CRC screening tests, such as FIT, in areas where health systems, surgical services, and resources are limited in capacity or not available at all. Stool-based tests may also address issues of cost, as they are more affordable than colonoscopy. Alternatively, facilities that use less than 100% of their colonoscopy capacity have the potential for pursuing interventions to maximize use of existing CRC screening services.

## Action

The North Dakota Colorectal Cancer Roundtable used this map to initiate discussions around increasing state capacity for CRC screening and improving access to screening services. Maps like this that describe the status of particular health services at a statewide level are valuable for generating discussion and initiating exploration into further analyses that may guide strategic planning for collective health prevention and promotion efforts.

Additional investigation into barriers to CRC screening, including access (regarding distance and travel), socioeconomic considerations, insurance coverage, and perceptions of various screening tests, is necessary to increase overall CRC screening rates and reduce the burden of CRC mortality on North Dakotans.
